# Complete chloroplast genome sequence of *Michelia champaca* var*. champaca* Linnaeus, an ornamental tree species of Magnoliaceae

**DOI:** 10.1080/23802359.2020.1790314

**Published:** 2020-07-15

**Authors:** Shaoyu Chen, Tao Wu, Yupin Fu, Jiabo Hao, Huifen Ma, Yunfeng Zhu, Yongkang Sima

**Affiliations:** Yunnan Academy of Forestry and Grassland Science, Kunming, People's Republic of China

**Keywords:** Magnoliaceae, *Michelia champaca* var. *champaca* Linnaeus, complete chloroplast genome, phylogenetic analysis

## Abstract

*Michelia champaca* var*. champaca* is an ornamentally important tree in Magnoliaceae. The paper reported the complete chloroplast genome (cpDNA) of *M. champaca* var*. champaca* and its basic annotated information. The size of cpDNA is 160,008 bp, with a typical quadripartite structure of a large single-copy (LSC) region of 88,037 bp and a small single-copy (SSC) region of 18,809 bp separated by a pair identical inverted repeat regions (IRs) of 26,581 bp each. The genome contained 131 genes (113 unique), including 86 protein-coding genes (80 unique), 37 tRNA genes (29 unique), and eight rRNA genes (four unique). Phylogenetic analysis showed that *M. champaca* var*. champaca* is affinal to *M. baillonii* and they form a nomophyletic group with other eight *Michelia* species. This *Michelia* clade is sister to the *Aromadendron cathcartii* clade with high support. All genera mentioned in this analysis are nomophyletic under the system of Magnoliaceae by Sima and Lu.

*Michelia champaca* Linnaeus is the type species of the genus *Michelia* Linnaeus in the family Magnoliaceae (Sima and Lu 2012). It has got two varieties, var. *champaca* Linnaeus and var. *pubinervia* (Blume) Miquel. The latter variety occurs in China (S Xizang, S and SW Yunnan), Cambodia, India, Indonesia, Laos, Malaysia, Myanmar, Nepal, Thailand, and Vietnam (Xia *et al*. 2008; Nooteboom and Chalermglin 2009). *Michelia champaca* var. *champaca* Linnaeus is distributed in China (SE Xizang, S and SW Yunnan), Bangladesh, India, Malaysia, Myanmar, Nepal, Pakistan, and Sri Lanka and widely cultivated as a good ornamental species in Southern China and Southeastern Asia, which is probably originally from India (Xia *et al*. 2008; Kundu 2009; Nooteboom and Chalermglin 2009; Jiang *et al*. 2013). It is grown as a plant for perfume and used medicinally (Khan *et al*. 2002; Jiang *et al*. 2012; Ma *et al*. 2012; Sinha and Varma 2017). Its flowers are used as nice perfume trinkets by women in many places in China (Jiang *et al*. 2013). However, there has been no report on chloroplast genome information of *M. champaca* var. *champaca* Linnaeus until now.

The complete sequence of chloroplast genome of *M. champaca* var*. champaca* Linnaeus was reported in this study. Genomic DNA was extracted using DNA Plantzol Reagent (Invitrogen, Carlsbad, CA, USA) from the fresh leaves of *M. champaca* var*. champaca* Linnaeus collected from Kunming Arboretum, Yunnan Academy of Forestry and Grassland Science, Yunnan Province of China (25°9'8″ N, 102°44'46″ E). The sheets of vouchered specimen (Y. K. Sima and S. Y. Chen 99278) are deposited at the herbaria, YAF and YCP.

Total genome DNA of *M. champaca* var*. champaca* Linnaeus was sequenced by Illumina HiSeq Sequencing System (Illumina, San Diego, CA) and shotgun library was constructed. About 2.0 G pair-end (150 bp) raw sequence data were obtained and the low-quality sequences were filtered through CLC Genomics Workbench v8.0 (CLC Bio, Aarhus, Denmark) to get high-quality clean reads. NOVO Plasty software (Dierckxsens *et al*. 2017) was used to align and assemble cp genome with *Pachylarnax sinica* (Y. W. Law) N. H. Xia and C. Y. Wu (JX280400) served as the reference. The genome was automatically annotated using CpGAVAS (Liu *et al*. 2012) and then adjusted and confirmed with Geneious 9.1 (Kearse *et al*. 2012). The annotated genomic sequence was submitted to GenBank under Accession Number of MT269873 (https://www.ncbi.nlm.nih.gov/nuccore/MT269873.1). To better determine the phylogenetic position of *M. champaca* var*. champaca* Linnaeus, the complete chloroplast genome sequences of other thirty-five species of the subfamily Magnolioideae from NCBI were aligned using MAFFT v. 7 (Sima and Lu 2012; Katoh and Standley 2013; Sima *et al*. 2014). Based on the system of Magnoliaceae by Sima and Lu (2012), two species of the subfamily Liriodendroideae, *Liriodendron chinense* (Hemsley) Sargent (KU170538) and *Liriodendron tulipifera* Linnaeus (DQB99947) were served as the outgroup. The maximum likelihood (ML) tree was reconstructed with RAxML (implemented in Geneious ver.10.1 http://www.geneious.com, Kearse *et al*. 2012) and bootstrap probability values were calculated from 1000 replicates.

The complete cp genome of *M. champaca* var*. champaca* Linnaeus is 160,008 bp in length, with a large single-copy (LSC) region of 88,037 bp and a small single-copy (SSC) region of 18,809 bp separated by a pair of identical inverted repeat regions (IRs) of 26,581 bp each. The cp genome contained 131 genes (113 unique), including 86 protein-coding genes (80 unique), 37 tRNA genes (29 unique), and eight rRNA genes (four unique). The result of phylogenetic analysis showed that *M. champaca* var*. champaca* Linnaeus is affinal to *Michelia baillonii* (Pierre) Finet and Gagnepain (MK782763) and they form a nomophyletic group with other eight species of the genus *Michelia* Linnaeus (Figure 1). This clade of the genus *Michelia* Linnaeus is sister to the clade of *Aromadendron cathcartii* (J. D. Hooker and Thomson) Sima and S. G. Lu with high support. All genera mentioned in this analysis are nomophyletic under the system of Magnoliaceae by Sima and Lu (2012). The study would provide effective new molecular data for evolutionary and phylogenetic analysis of Magnoliaceae.

**Figure 1.**
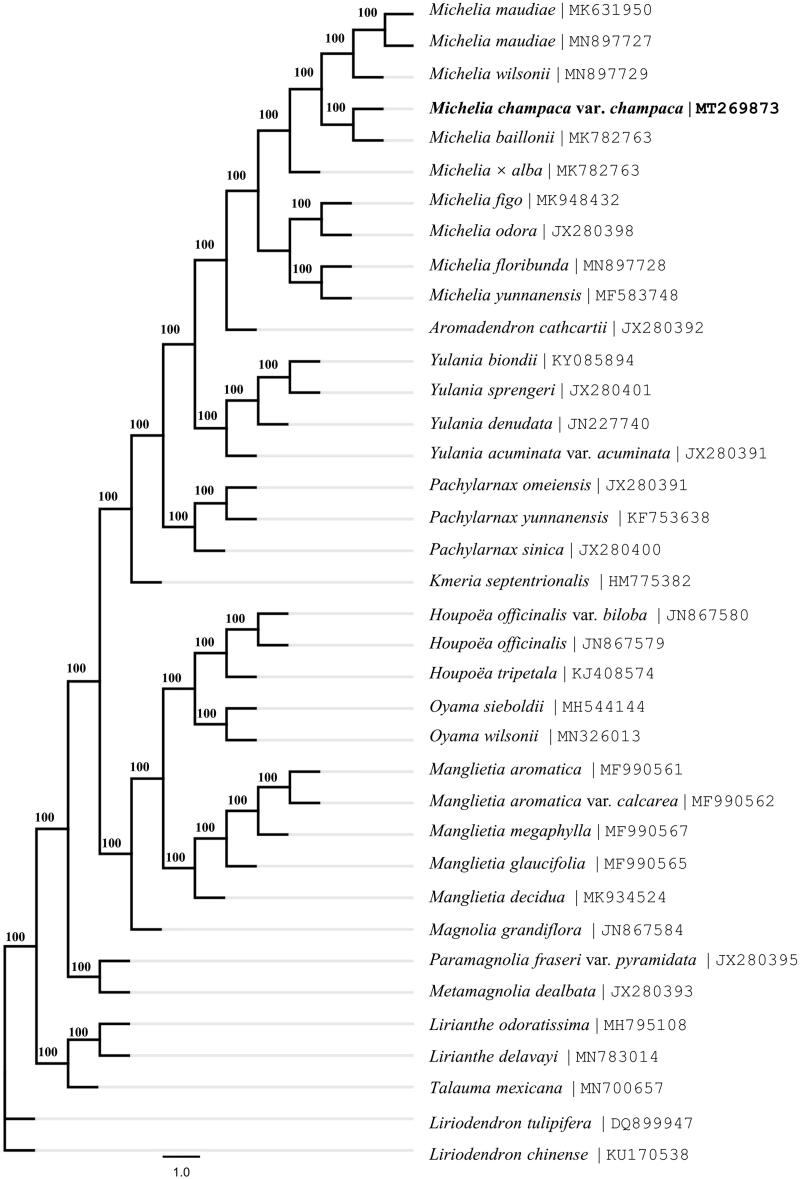
The maximum-likelihood tree based on the chloroplast genomes of 35 species of Magnolioideae and 2 species of Liriodendroideae in the family Magnoliaceae. Bootstrap values (1000 replicates) are shown at the nodes.

## Data Availability

The sheets of vouchered specimen, Y. K. Sima and S. Y. Chen 99278, have been stored at the herbaria, YAF and YCP. The data of complete chloroplast genome sequence of *Michelia champaca* var. *champaca* Linnaeus have been deposited into NCBI databases and the accession number is MT269873 (https://www.ncbi.nlm.nih.gov/nuccore/MT269873.1). The data above should only be shared if it is ethically correct to do so, where this does not violate the protection of human subjects, or other valid ethical, privacy, or security concerns.
